# Acute paraplegia caused by thromboembolism from an infrarenal abdominal aortic aneurysm following colonoscopy

**DOI:** 10.1097/MD.0000000000046995

**Published:** 2026-01-02

**Authors:** Il Soon Jung

**Affiliations:** a Department of Internal Medicine, Chungbuk National University Hospital, Cheongju, Republic of Korea; b Department of Internal Medicine, Chungbuk National University College of Medicine, Cheongju, Republic of Korea.

**Keywords:** abdominal aortic aneurysm, adverse event, colonoscopy, paraplegia, thromboembolism

## Abstract

**Rationale:**

Colonoscopy is a widely used diagnostic and therapeutic procedure with a low risk of serious adverse events. Acute bilateral lower-limb paralysis following colonoscopy is extremely rare. Acute limb ischemia due to thromboembolism from abdominal aortic aneurysm (AAA) is a vascular emergency that requires prompt diagnosis and intervention. This is the report of a unique case of acute limb ischemia due to thromboembolism associated with infrarenal AAA following colonoscopy. This case report aims to raise clinical awareness of the potential risk of thromboembolic complications after colonoscopy. Further, this case suggests a potential mechanism in which hemodynamic alterations or periprocedural factors, such as dehydration associated with bowel preparation, may have contributed to thrombus dislodgement and subsequent embolization.

**Patient concerns:**

A 67-year-old man with acute paraplegia was referred to our emergency department following colonoscopy.

**Diagnoses:**

The patient was diagnosed with acute thromboembolism associated with an infrarenal AAA, resulting in paraplegia following colonoscopy, as confirmed by computed tomography angiography.

**Interventions:**

The patient underwent emergent aorto-bifemoral bypass, bilateral lower extremity thrombectomy, and left femoral artery-to-distal superficial femoral artery bypass surgery.

**Outcomes:**

Although revascularization was technically successful, the patient died of massive reperfusion injury.

**Lessons:**

Acute paraplegia caused by thromboembolism from an infrarenal AAA following colonoscopy is extremely rare but potentially fatal. Awareness of this potential adverse event after colonoscopy is critical to avoid severe outcomes and improve patient prognoses.

## 1. Introduction

Colonoscopy is one of the most widely used diagnostic methods worldwide for the screening, diagnosis, and treatment of colorectal diseases. Colonoscopy is considered a relatively low-risk procedure; however, various complications have been reported in approximately 2.8 cases per 1000 procedures.^[1]^ The incidence rate of serious complications was reported to be a low 0.28% in a systematic analysis of 12 articles that involved 57,742 individuals who underwent screening colonoscopy.^[2]^ Overall, procedure-related mortality has been reported to be as low as 0.09%.^[1]^

To the best of my knowledge, unexpected thromboembolism of an infrarenal abdominal aortic aneurysm (AAA) (not previously diagnosed) following colonoscopy has not been described in the scientific literature. This is the 1st report of a case of acute paraplegia due to thromboembolism associated with an infrarenal AAA following colonoscopy.

## 2. Case presentation

A 67-year-old man with acute-onset paraplegia was referred to our emergency department within 1 hour of awakening from sedation after undergoing a routine screening colonoscopy under conscious sedation at a private clinic. Four small polyps had been identified and removed using biopsy forceps. The procedure was completed with no remarkable events during the colonoscopy and without any immediate complications.

Immediately after colonoscopy, the patient complained of an inability to move both lower limbs and sensory loss, but without associated pain. Consequently, he was referred to our emergency department. The patient had a past medical history of hypertension and a herniated intervertebral disc. His vital signs showed a blood pressure of 185/93 mm Hg, heart rate of 99 beats/min, respiratory rate of 13 breaths/min, and body temperature of 36.8°C. Physical examination and neurological examination revealed complete absence of motor function in the bilateral lower extremities, and sensory loss was noted below the L2 dermatome. Deep tendon reflexes were hyperactive, and the plantar responses were bilateral flexors. Initially, no discoloration of the lower extremities was observed bilaterally. Laboratory test results are detailed in Table 1. The tests revealed markedly elevated D-dimer and fibrin degradation product levels. However, these findings were nonspecific. Also, normal blood urea nitrogen, creatinine, serum sodium, and osmolality suggest that clinically significant dehydration was unlikely.

**Table 1:** Patient’s laboratory test results.

Parameter	Result	Reference range
WBC	13,820/μL	4000–10,000/μL
Neutrophils (%)	77.9%	40–75%
Hemoglobin	15.5 g/dL	13–17 g/dL
Platelet count	107 × 10³/μL	150–400 × 10³/μL
hsCRP	0.13 mg/dL	<0.5 mg/dL
BUN	13.7 mg/dL	8–20 mg/dL
Serum creatinine	0.8 mg/dL	0.6–1.3 mg/dL
Serum sodium	142 mEq/L	135–145 mEq/L
Serum osmolality	296 mOsm/kg	275–295 mOsm/kg
PT (INR)	13.5 s (1.24)	10–13 s (0.8–1.2)
aPTT	25.9 s	25–35 s
FDP	639 µg/mL	<5 µg/mL
D-dimer	4.5 mg/L	<0.5 mg/L

aPTT = activated partial thromboplastin time, BUN = blood urea nitrogen, FDP = activated partial thromboplastin time, hsCRP = high-sensitivity C-reactive protein, INR = International Normalized Ratio, PT = prothrombin time, WBC = white blood cell count.

**Figure 1.**
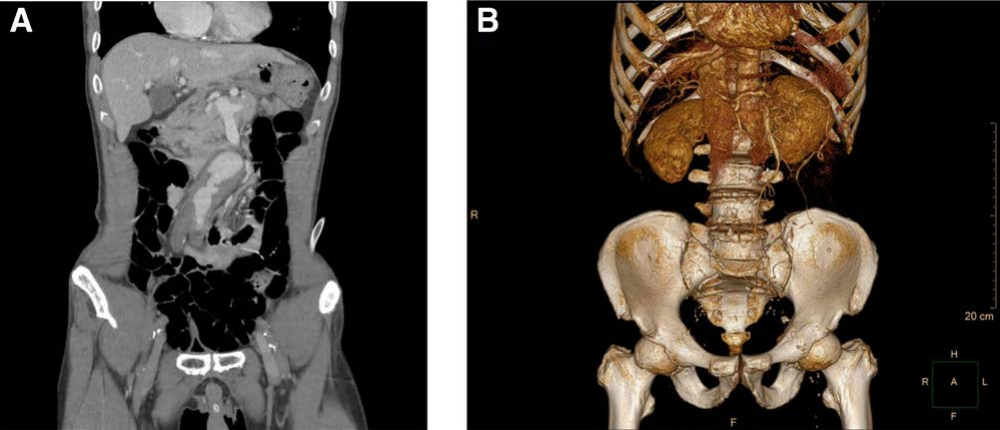
CTA showing an infrarenal AAA with thrombus formation measuring approximately 42 mm in diameter and total occlusion of the common bilateral iliac arteries. (A) Coronal image; (B) 3D reconstruction. AAA = abdominal aortic aneurysm, CTA = computed tomography angiography.

**Figure 2.**
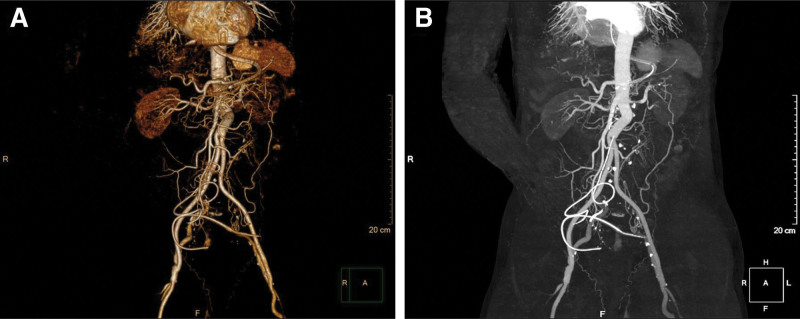
Successful revascularization achieved through a combination of aorto-bifemoral bypass, bilateral lower extremity thrombectomy, and construction of a femoral-to-distal SFA bypass. (A) Volume rendering of CTA; (B) Maximum intensity projection of CTA. SFA = superficial femoral artery, CTA = computed tomography angiography.

Based on the patient’s clinical history, laboratory tests and physical examination findings, acute spinal cord compression was suspected, and spinal magnetic resonance imaging was performed. While the patient was awaiting spinal magnetic resonance imaging, point-of-care ultrasound was performed, which revealed the presence of an AAA. Considering the possibility of acute thromboembolism in both lower limbs, computed tomography angiography was promptly performed. Computed tomography angiography showed an infrarenal AAA with thrombus formation measuring approximately 42 mm in diameter and nonvisible bilateral common iliac arteries (Fig. 1). These findings suggest that thrombi originating from the aortic aneurysm obstructed the arteries supplying both lower extremities, leading to acute lower-limb ischemia. Emergency surgery was initiated within 5 hours of the patient’s arrival at the emergency department. Intraoperative findings revealed an infrarenal AAA with total occlusion of both common iliac arteries. The stenotic segment of the left femoral artery was surgically opened, and thrombus removal was performed using a Fogarty catheter. Successful revascularization was achieved by constructing a bypass from the femoral artery-to-distal superficial femoral artery (Fig. 2). Postoperatively, perfusion to both lower extremities was restored and an improvement in limb coloration was observed. The duration from the onset of acute limb ischemia to successful reperfusion was 11 hours.

After colonoscopy, the patient developed acute bilateral lower-limb ischemia, resulting in paraplegia. Emergency surgery was performed to restore blood flow; however, because reperfusion did not occur within the critical 6-hour window, postoperative reperfusion injury occurred. Subsequently, wound deterioration and muscle necrosis progressed, leading to sepsis. Despite aggressive medical and surgical management, the patient ultimately did not survive due to multisystem organ failure secondary to sepsis.

## 3. Discussion

Acute paraplegia due to thromboembolism associated with an infrarenal AAA following colonoscopy is extremely rare and represents a potentially life-threatening adverse event. Sudden thromboembolism in an AAA is uncommon. Shumacker et al published the first report of AAA thrombosis in 1959.^[3]^ This has been reported to occur in approximately 0.6% to 2.8% of all AAA cases that are treated surgically.^[4–6]^

Several hypotheses have been proposed in the literature regarding the development of acute thromboembolism associated with an AAA. These include conditions related to surgical or abdominal manipulation; trauma; an acute low-flow state, such as hypotension, dehydration, fever, or hemorrhage; a hypercoagulable state; displacement of fragments of the mural thrombus inside the aneurysm resulting in an occlusion of the outflow tract of the aneurysm; and cardio-aortic embolization secondary to arrhythmia.^[6,7]^ External abdominal compression during colonoscopy may provoke plaque instability and thrombus dislodgement in patients with undiagnosed vascular pathologies such as an AAA or atherosclerosis of the arteries. In this patient, 1 possible explanation involves local plaque rupture at the aneurysm site triggered by abdominal manipulation, particularly external abdominal compression, leading to dissection and fragmentation of the atheromatous thrombus adherent to the aneurysm wall. This hypothesis is analogous to the rare but documented cases of stroke following carotid vibro-compressive maneuvers, carotid sinus massage, and routine carotid ultrasonography in patients with underlying carotid atherosclerosis or ulcerated carotid plaques.^[8,9]^ Specific colonoscopic maneuvers, such as the slide by advancement, alpha maneuver, hooking the flexure, and straightening the sigmoid loop during colonoscopy have been associated with excessive traction, distension, and vibration.^[10]^

Another contributing factor may be the relative dehydration induced by bowel preparation. Dehydration, a common complication of bowel preparation, likely exacerbates this risk by increasing the blood viscosity and promoting clot formation.^[11,12]^ Olinic et al reported that patients with cardiovascular disease (including cardiac valvular disease, aortic and cerebral atherosclerosis, arrhythmia, and aneurysm) frequently have concomitant peripheral arterial disease. In such patients, peripheral arterial damage can lead to thromboembolic events, which may occur spontaneously or be triggered by an external injury or internal hemodynamic changes.^[13]^

In this report, colonoscopy may be a triggering factor. During the procedure, abdominal compression, increased intra-abdominal pressure, and bowel distension could have altered the hemodynamics within the infrarenal AAA. When combined with dehydration, these changes may have promoted mural thrombus formation, plaque rupture, or detachment of a preexisting thrombus.

In general, acute paraplegia has several differential diagnoses. In a case of sudden bilateral lower-limb paralysis occurring in a patient with an underlying condition, the differential diagnoses should include neurological causes such as stroke, spinal cord injury, Guillain–Barré syndrome, and multiple sclerosis. Inflammatory or infectious conditions, such as transverse myelitis and infectious myelitis, should also be considered. Other possibilities include neuromuscular disorders such as myasthenia gravis; vascular causes such as thrombosis or peripheral artery disease; and metabolic or endocrine disturbances, including hypokalemia or hyperthyroidism.

In this case, the patient was unaware of his underlying condition, and the emergency physician initially considered acute spinal cord compression because of the patient’s history of lower back pain and herniated disc. This led to a slight delay in the diagnosis. However, at our emergency department, the emergency physician routinely performed abdominal ultrasound, enabling a relatively early detection of an AAA. Urgent thrombectomy and AAA reconstruction were subsequently performed. However, the reperfusion time exceeded the golden window of 6 hours, resulting in reperfusion injury, which ultimately led to the patient’s death.

This case describes a unique presentation and temporal association of acute paraplegia caused by thromboembolism with colonoscopy (a routine and generally safe procedure). To the best of my knowledge, there are no previous reports of acute paraplegia caused by thromboembolism from an infrarenal AAA immediately after colonoscopy. In general, thorough preprocedural evaluations are conducted in patients with known comorbidities to minimize procedural risks. However, complications may occur in elderly or vulnerable patients who are unaware of their lifestyle-related atherosclerotic risk factors. Therefore, this case underscores the importance of heightened clinical vigilance and individualized risk assessment of patients undergoing seemingly low-risk procedures such as colonoscopy.

## 4. Conclusions

This is a unique case of acute limb ischemia due to thromboembolism associated with an infrarenal AAA following colonoscopy. Several plausible mechanisms for acute limb ischemia following colonoscopy have been proposed. Furthermore, the importance of individualized risk assessment before routine procedures in elderly patients is highlighted. Clinicians should be aware of rare but potentially devastating complications, such as acute limb ischemia, that may occur even after an uneventful colonoscopy.

## Author contributions

**Conceptualization:** Il Soon Jung.

**Writing – original draft:** Il Soon Jung.

**Writing – review & editing:** Il Soon Jung.
